# AI4AVP: an antiviral peptides predictor in deep learning approach with generative adversarial network data augmentation

**DOI:** 10.1093/bioadv/vbac080

**Published:** 2022-10-26

**Authors:** Tzu-Tang Lin, Yih-Yun Sun, Ching-Tien Wang, Wen-Chih Cheng, I-Hsuan Lu, Chung-Yen Lin, Shu-Hwa Chen

**Affiliations:** Institute of Information Science, Academia Sinica, Taipei 115, Taiwan; Institute of Information Science, Academia Sinica, Taipei 115, Taiwan; Graduate Institute of Biomedical Electronics and Bioinformatics, College of Electrical Engineering and Computer Science, National Taiwan University, Taipei 106, Taiwan; Institute of Information Science, Academia Sinica, Taipei 115, Taiwan; Institute of Information Science, Academia Sinica, Taipei 115, Taiwan; Institute of Information Science, Academia Sinica, Taipei 115, Taiwan; Institute of Information Science, Academia Sinica, Taipei 115, Taiwan; Institute of Fisheries Science, National Taiwan University, Taipei 106, Taiwan; Genome and Systems Biology Degree Program, National Taiwan University, Taipei 106, Taiwan; TMU Research Center of Cancer Translational Medicine, Taipei Medical University, Taipei 110, Taiwan

## Abstract

**Motivation:**

Antiviral peptides (AVPs) from various sources suggest the possibility of developing peptide drugs for treating viral diseases. Because of the increasing number of identified AVPs and the advances in deep learning theory, it is reasonable to experiment with peptide drug design using *in silico* methods.

**Results:**

We collected the most up-to-date AVPs and used deep learning to construct a sequence-based binary classifier. A generative adversarial network was employed to augment the number of AVPs in the positive training dataset and enable our deep learning convolutional neural network (CNN) model to learn from the negative dataset. Our classifier outperformed other state-of-the-art classifiers when using the testing dataset. We have placed the trained classifiers on a user-friendly web server, AI4AVP, for the research community.

**Availability and implementation:**

AI4AVP is freely accessible at http://axp.iis.sinica.edu.tw/AI4AVP/; codes and datasets for the peptide GAN and the AVP predictor CNN are available at https://github.com/lsbnb/amp_gan and https://github.com/LinTzuTang/AI4AVP_predictor.

**Supplementary information:**

Supplementary data are available at *Bioinformatics Advances* online.

## 1 Introduction

A viral pandemic has substantial impacts on every aspect of our lives. Despite the obvious need, the treatment options available for viral diseases other than supportive care are limited. Developing a new high-efficacy drug for a viral pathogen, such as Tamiflu for preventing influenza virus propagation and Acyclovir for treating vesicular stomatitis virus infection, is a difficult task, as has been learned from the pandemic of coronavirus disease 2019 (COVID-19), monkeypox and other viral pathogens on the horizon. Antiviral reagents often have systemic side effects or low efficacy because drug-resistant strains of viruses emerge (Agarwal and Gabrani, 2021). Antiviral peptides (AVPs) are effective against re-emerging and drug-resistant viruses (Mahendran *et al.*, 2020). They are natural and peptidase biodegradable and have low toxicity (Boas *et al.*, 2019). The existence of AVPs indicates that synthetic peptides have the potential to combat viral diseases. However, randomly generating sequences for AVP screening is not a cost-effective approach.

Several papers have discussed the development of artificial peptide sequences with AVP activity. Thakur *et al.* (2012) proposed an AVP prediction algorithm based on a model derived from experimentally validated positive and negative data sets and wrapped the model into the web tool AVPpred (Thakur *et al.*, 2012). The dataset from that study was used in other *in silico* peptide designs, such as AntiVPP 1.0 (Beltran Lissabet *et al.*, 2019), Meta-iAVP (Schaduangrat *et al.*, 2019) and FIRM-AVP (Chowdhury *et al.*, 2020). In these designs, amino acid composition, amino acid sequences, motif structures and physicochemical properties are considered features. AVP predictors were built using machine learning methods such as random forest and support vector machine (Supplementary Table S1).

In the present study, we introduce AI4AVP, an AVP predictor. With the most up-to-date AVP set, a deep learning model based on a convolutional neural network (CNN) was trained and compared with other AVP predictors. We used PC6 encoding (Lin *et al.*), a protein-encoding method based on six physicochemical properties, to transform sequential data into matrices. We developed a generative adversarial network (GAN) model for AVP drug development based on our previous work. GAN has been applied to various bioinformatic problems involving protein or DNA design (Linder *et al.*, 2020; Liu *et al.*, 2019, 2021; Wang *et al.*, 2020).

We used a peptide generator for data augmentation to increase the input data size without disturbing the positive–negative balance. The final trained CNN models are accessible as a web tool and can be used to evaluate the AVP potency of user-submitted sequences. AI4AVP can help AVP researchers evaluate the antiviral potential of unknown peptides.

## 2 Methods

### 2.1 Data collection and preprocessing

AVP sequence sets were collected from APD3 (Wang *et al.*, 2016), DRAMP (Kang *et al.*, 2019), YADAMP (Piotto *et al.*, 2012), DBAASP (Pirtskhalava *et al.*, 2020), CAMP (Waghu *et al.*, 2016) and AVPdb (Qureshi *et al.*, 2014) (Supplementary Table S2). Sequences containing the keyword ‘anti-viral’ and its synonyms were collected and cleaned to create a non-redundant set. Sequences were excluded if they contained unusual letters (e.g., ‘B’, ‘Z’, ‘U’, ‘J’, ‘O’, ‘X’, ‘I’, ‘n’, or ‘−’) or if their length was not between 10 and 50 residues. We used CD-HIT (Huang *et al.*, 2010) for reported representatives of highly similar sequences with 95% identity. Finally, a cleaned AVP set (AVP_fullset) composed of 2934 sequences was collected.

We randomly collected peptide sequences unrelated to antiviral function from the UniProt/SwissProt database (UniProt Consortium, 2021) to construct a negative dataset and generate artificial sequences. Briefly, short non-AMP peptides are defined as peptides not tagged with keywords related to antimicrobial peptide function (e.g. ‘anti-microbial’, ‘anti-viral’, ‘antibiotic’, ‘amphibian defense peptide’ or ‘antiviral protein’) and with the length between 10 and 50 residues—were obtained from Swiss-Prot. An equal number (*n* = 8592) of random peptide sequences with a length between 10 and 50 residues was appended to create the negative set (*n* = 17 184) (Supplementary Table S2).

For model training, we used 90% of the cleaned AVPs and an equal number of randomly chosen peptides from the negative dataset to create a balanced input named AVP_training (2641 positives + 2641 negatives). For model validation, we used the spare AVPs plus an equal number of sequences from the negative dataset to create AVP_testing (293 positives + 293 negatives).

We also collected and revised the dataset of Thakur *et al.* (2012), denoted 2012_training. The dataset comprises 506 AVPs and 506 non-AVPs and has been used in the training of predictors such as AVPpred (Thakur *et al.*, 2012), AntiVPP 1.0 (Beltran Lissabet *et al.*, 2019), Meta-iAVP (Schaduangrat *et al.*, 2019) and FIRM-AVP (Chowdhury *et al.*, 2020). Here, we used Python to process the data and build our models. We also implemented Keras, a high-level API of Tensorflow v2.10.0, to shape our deep learning model and the Scikit-learn package to construct the random forest models and support vector machine.

### 2.2 Data augmentation by GAN

We trained a generative model with AVP_fullset (*n* = 2934) to generate AVP-like sequences. As shown in Supplementary Figure S1, the latent noise vectors were transformed into generated AVPs through the *generator* network. The *discriminator* network then assessed the real and generated AVPs before updating the model weights through backpropagation. These two competing neural networks were modified in each iteration of training (Supplementary Fig. S1). We used WGAN-GP (Gulrajani *et al.*, 2017), a GAN with higher stability and less severe mode collapse problems compared with the original GAN (Goodfellow *et al.*, 2014). For a given pair of a generator G and a discriminator D, the training process is a min-max game that maximizes the probability of correctly detecting training data and minimizes the difference between the training data and the generated set. The loss function of WGAN-GP is defined as follows:

$$L\;=E_{\tilde{x}∼P_{g}}\left[D\left(\tilde{x}\right)\right]{\;-\;E}_{x∼P_{r}}\left[D\left(x\right)\right]+\;\lambda E_{\tilde{x}∼P_{\tilde{x}}}[{{(\left\|\nabla_{\tilde{x}}D\left(\tilde{x}\right)\right\|}_{2}-\;1)}^{2}]$$

In the equation, $P_{r}$ and $P_{g}$ are the data distributions from the training set and generated set, respectively; x is the data sampled from $P_{r}$, and $\tilde{x}$ is the data sampled from $P_{g}$. $P_{\tilde{x}}$ represents the uniform *y* between $P_{r}$ and $P_{g}$, and λ is a penalty coefficient. By adding a gradient penalty ($\lambda E_{\tilde{x}∼P_{\tilde{x}}}[{{(\left\|\nabla_{\tilde{x}}D\left(\tilde{x}\right)\right\|}_{2}-\;1)}^{2}]$) in Wasserstein GAN (WGAN) (Arjovsky *et al.*, 2017), the Lipschitz continuity is achieved. Unlike other GANs in which batch normalization is used to help stabilize the training, layer normalization is employed in WGAN-GP to fit the gradient penalty by processing each input independently. Our previous study used WGAN-GP to generate peptides (Lin *et al.*, 2021). The peptide generator/discriminator GAN implementation is available on GitHub (https://github.com/lsbnb/amp_gan).

Thousands of AVP-like sequences were generated. Finally, we built a hybrid dataset (AVP+GAN_training) composed of 16 995 positives from AVP_training and GAN-generated AVP-like sequences and 16 995 randomly chosen negatives from the negative dataset.

### 2.3 Protein-encoding method

We used the protein-encoding method *PC6* (Lin *et al.*) to transform peptide sequences into numeric matrices (Supplementary Fig. S2). This encoding method can express the arrangement of residues and the physicochemical properties of amino acids, thus offering essential features for model training. Another encoding method used in this study was the *descriptor encoding* used in ENNAVIA (Timmons and Hewage, 2021). We implemented the *descriptor encoding* method as described in the paper. Briefly, we calculated global physicochemical descriptors using the modlAMP package (Müller *et al.*, 2017) and composition descriptors—such as the amino acid composition, pseudo amino acid composition, AA index, and dipeptide composition—using the iFeature package (Chen *et al.*, 2018).

### 2.4 AVP predictor model construction

**Fig. 1.**
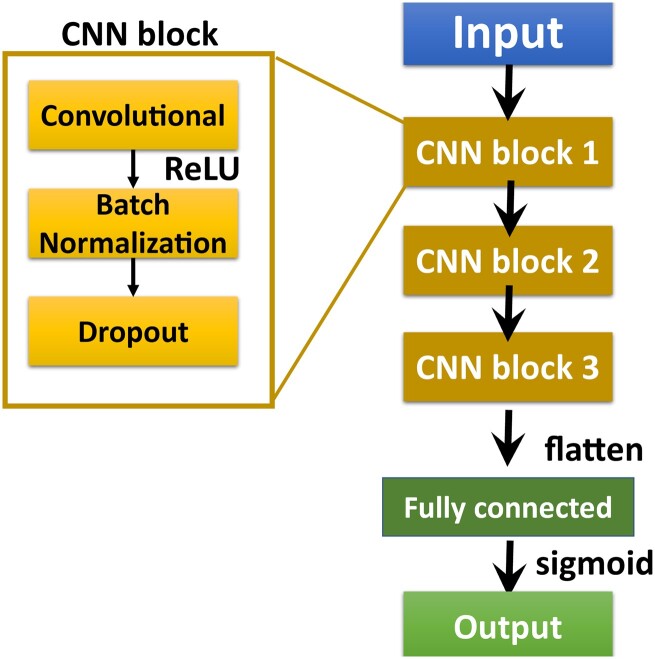
Architecture of the AI4AVP model. The encoded peptide matrix serves as the input that passes through the three CNN blocks. The fully connected layer with a sigmoid activation function transforms the vector into a value between 0 and 1 to produce the model’s output, the prediction.

We implemented the AVP predictor deep learning model in Python using tf.keras, a high-level API from Tensorflow. The model was constructed on a three-CNN-block architecture. Each CNN block comprised a convolutional layer [filters: (64, 32, 16), kernel_size: (8, 8, 8)] with a rectified linear activation function, a batch normalization layer, and a dropout layer [rate: (0.5, 0.5, 0.5); Fig. 1]. The output value, between 0 and 1, was produced by a fully connected layer (unit: 1) with a sigmoid activation function. We set the batch size of the validation dataset to 1000. The validation loss of every epoch during model training was evaluated, and the training was stopped when the model’s performance had stabilized. The model with the lowest validation loss was saved as the optimal model.

We also constructed models using conventional machine learning schemes, such as random forest and support vector machine. The algorithms were implemented using the Scikit-learn package. Finally, we compared the model’s performance with and without GAN data augmentation for every model we constructed.

### 2.5 Model evaluation and performance measures

Three model training datasets were used in this study. Because of the limitations of model retraining, we changed the training set to ensure a fair comparison. We trained our model on 2012_training [506 AVPs and 506 non-AVPs, used in other AVP predictors (Beltran Lissabet *et al.*, 2019; Chowdhury *et al.*, 2020; Schaduangrat *et al.*, 2019; Thakur *et al.*, 2012)] and compared the predictor’s performance with that of other AVP predictors using the same dataset. We then trained another AVP_training (2641 positives + 2641 negatives) to construct a predictor using the updated information. To fully utilize the information in negative non-AVP sequences, we trained a GAN model for data augmentation, as previously described, and then used the hybrid AVP+GAN_training (16 995 positives + 16 995 negatives) to include as much information as possible in the AVP predictor model training.

We evaluated the model’s performance in terms of accuracy, precision, sensitivity, specificity, and the Matthews correlation coefficient (MCC). These were calculated as follows:

$$\mathrm{accuracy}=\frac{TP+TN}{TP+FP+TN+FN}\times 100$$

$$\mathrm{precision}=\frac{TP}{TP+FP}\;\times 100$$

$$\mathrm{sensitivity}=\frac{TP}{TP+FN}\times 100$$

$$\mathrm{specificity}=\frac{TN}{TN+FP}\times 100$$

$$\mathrm{MCC}=\;\frac{\left(TP\times TN\right)-(FP\times FN)}{\sqrt{\left(TP+FP)(TP+FN)(TN+FP)(TN+FN\right)}}$$

where *TP* represents the number of true positive predictions, *TN* is the number of true negative predictions, *FP* is the number of false positive predictions and *FN* is the number of false negative predictions.

### 2.6 AI4AVP website

**Fig. 2.**
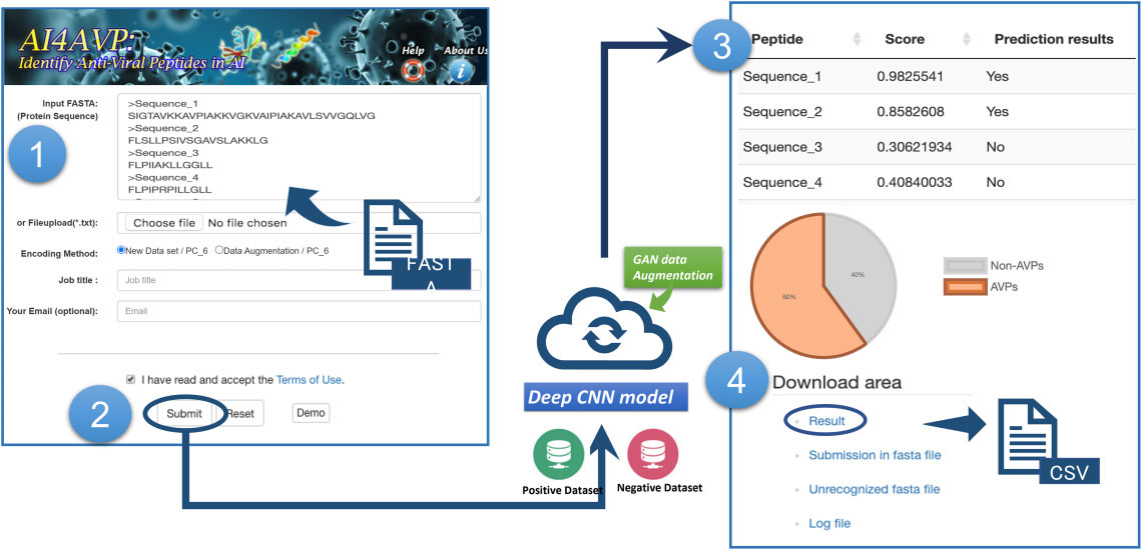
AI4AVP web server workflow. (1 and 2) AI4AVP interface. The input of AI4AVP is peptide sequences in FASTA format, submitted in either copy-paste or batch execution in a file upload manner with the selected encoding method. Users can wait for the server’s response or supply a valid email to retrieve the result upon job completion notification. (3) The window clip of the result page. (4) The predictions of each peptide are summarized in a CSV file that is downloadable from the ‘Download area.’.

We developed AI4AVP, a web server that enables users to employ the deep learning model constructed in this study (Fig. 2). Users can submit their peptide sequences through a friendly interface, receive the calculated prediction results, and select the predictor trained using AVP_training or AVP+GAN _training. The input sequence should be in FASTA format with a minimal length of 10 residues. For inputs longer than 50 residues, AI4AVP will chop the input into multiple strings (window size: 50, step: 25) before running the prediction.

## 3 Results

In this study, we introduced a new AVP predictor that employs a deep learning algorithm and is trained with updated data. An experimental approach was also attempted to recruit more information from the negative dataset without compromising the balance of the input training set (Supplementary Fig. S3). Table 1 presents the model’s performance when trained on three datasets: 2012_training, AVP_training and AVP+GAN_training. The neural network outperformed the other two tested algorithms in AVP_training and hybrid AVP+GAN_training. In the AVP+GAN_training, the precision and specificity of the CNN model were high, contributing to the final MCC being 0.68 rather than 0.65, as was achieved for the AVP_training. We also conducted a 10-fold cross-validation to evaluate the model’s stability, as shown in Supplementary Table S3. For all models, the standard deviation was low (<0.1). The 10-fold cross-validation result based on the model encoding PC6 on CNN with a new training dataset (AVP+ GAN_training) performs best on Accuracy and MCC by 0.94 and 0.88 in Supplementary Table S3, respectively. Compared to the same approach used on AVP_training, the value of Accuracy and MCC are 0.87 and 0.77 around. The same encoding model PC6 with CNN can benefit greatly from the sequences generated in wGAN-GP.

**Table 1.** Results of model evaluation with various datasets by the same testing set, AVP_testing^a^

Training set	Encoding method	Algorithm	Accuracy	Precision	Sensitivity	Specificity	MCC
2012_training^b^	PC6 encoding	CNN	0.55	0.61	0.29	0.82	0.12
AVP_training^b^	PC6 encoding	CNN	0.83	0.82	0.85	0.81	0.65
	PC6 encoding	RF	0.74	0.69	0.88	0.60	0.50
	PC6 encoding	SVM	0.73	0.68	0.86	0.59	0.47
	descriptor encoding^c^	CNN	0.78	0.77	0.81	0.76	0.57
AVP+ GAN_training^b^	PC6 encoding	CNN	**0.84**	**0.84**	0.85	**0.86**	**0.68**
	PC6 encoding	RF	0.74	0.69	0.85	0.62	0.49
	PC6 encoding	SVM	0.81	0.81	0.81	0.81	0.62
	descriptor encoding^c^	CNN	0.70	0.64	**0.94**	0.46	0.46

a **AVP_testing**: 293 positives + 293 negatives, selected from a clean AVP collection (AVP_fullset, 2934 positives + 2934 negatives).

^b^ **2012_training**: 506 positives + 506 negatives by Thakur *et al.* (2012); **AVP_training**: 2641 positives + 2641 negatives; **AVP+GAN_training**: 16 995 positives (from AVP_training and GAN-generated AVP-like sequences) + 16 995 negatives.

^c^ Descriptor encoding refers to the feature descriptor used in ENNAVIA (Timmons and Hewage, 2021), including composition and physicochemical scores. Each evaluation metric's best and second ones were marked in bold and underlined.

CNN, convolutional neural network; RF, random forest; SVM, support vector machine.

To make a fair comparison of the model’s performance, we trained another predictor in the same deep learning/protein encoding architecture on 2012_training, which is the same training data used by AVPpred, AntiVPP 1.0, Meta-iAVP, and FIRM-AVP. The validation was performed using AVP_test, which was not contaminated by the models’ training sets. According to Supplementary Table S4, all predictors, including ours, performed marginally, with an accuracy of ∼0.5 and an MCC of ∼0.1. All performance indices were significantly better than those obtained with the model trained on the new collection (AVP_training), which contained three times newer AVP sequences than 2012_training.

We compared the PC6 encoding method with the descriptor protein encoding method used in protein property prediction studies (Beltran Lissabet *et al.*, 2019; Chowdhury *et al.*, 2020; Schaduangrat *et al.*, 2019; Thakur *et al.*, 2012). We implemented the ENNAVIA (Timmons and Hewage, 2021) method involving both composition and physicochemical descriptors as features to train the neural network model. We discovered that the descriptive encoding performed similarly with our PC6 encoding when the model was trained with AVP_training. Still, the performance was lower in almost every measurement, except sensitivity, with AVP+GAN_training (Table 1).

## 4 Discussion

Predicting a peptide’s AVP potency based on its primary sequence is a typical binary classification problem in machine learning. The performance of machine learning depends on both the quality and quantity of data. After Thakur’s work, a few new AVPs were discovered. Using the same deep learning architecture, we found that increasing the amount of input data improves a classifier’s performance. Neural networks are generally better at constructing models from large datasets. Although we could not retrain the models of the AVP predictors from other studies, the experimental results indicated that a well-designed deep learning model (neural network) increasingly outperforms a random forest or support vector machine model as the size of the input dataset increases.

Because of the cost and labor involved in discovering and validating peptides, those clearly defined for a specific activity, such as the AVP we targeted in this study, are limited. We often had more negative data than positive data, but the concern of training data balance meant that some negative data could not be used. To increase the input data size without disturbing the positive–negative balance, we used GAN in the data augmentation process, generating AVP-like sequences as surrogates. We first initiated the generator model to create an AVP-like sequence based on real AVPs; this generator was reshaped during the model training process, making it a better generator. GAN augmentation also increased the positives, allowing almost all sequences in the negative set to be used in the AVP classifier model training, thereby improving the classifier’s robustness for peptide identification. As evidenced by Table 1, the model trained on the augmented dataset achieved higher accuracy and sensitivity and an overall better MCC score. We do not know why the CNN with descriptive encoding could not benefit from the increased dataset size.

## 5 Conclusions

We constructed AI4AVP, an AVP predictor, using a deep learning algorithm trained on the most up-to-date dataset and a previously published protein-encoding method (PC6). In our previous studies, we achieved data augmentation and developed a peptide GAN that can increase the amount of negative data used. This approach allowed us to use our training data while keeping the datasets balanced during model training. The AVP predictors trained on AVP_training and AVP+GAN_training is available as user-friendly web portal, AI4AVP, for predicting the antiviral ability of peptide sequences and accelerating the development of potential antiviral drugs.

## Supplementary Material

vbac080_Supplementary_Data

## Data Availability

The mySORT website with demo dataset and Docker image can be accessed for free at https://mysort.iis.sinica.edu.tw and https://hub.docker.com/r/lsbnb/mysort_2022.
