# Supplementary material for: AI4AVP: an antiviral peptides predictor in deep learning approach with generative adversarial network data augmentation
Source: Bioinform Adv. 2022 Oct 26;2(1):vbac080. doi: 10.1093/bioadv/vbac080 (PMC9710571; doi:10.1093/bioadv/vbac080)
Supplement: vbac080_Supplementary_Data [file vbac080_supplementary_data.pdf]

Supplementary Table 1. List of the predictors compared with AI4AVP in this study.

Predictors	Machine learning	Application type	Website
**AVPpred (1)**	Support Vector Machine	Web-server	http://crdd.osdd.net/servers/avppred/
**AntiVPP1.0 (2)**	Random Forest	Source code	https://github.com/bio-coding/AntiVPP
**Meta-iAVP (3)**	Ensemble Model	Web-server	http://codes.bio/meta-iavp/
**FIRM-AVP (4)**	Support Vector Machine	Web-server	https://msc-viz.emsl.pnnl.gov/AVPR/
**AI4AVP (this study)**	**Deep learning (CNN)**	Web-server	https://axp.iis.sinica.edu.tw/AI4AVP/

Supplementary Table 2. List of databases we collected data from.

Database	Label	Website
**APD3 (5)**	Positive	https://wangapd3.com/main.php
**DRAMP (6)**	Positive	http://dramp.cpu-bioinfor.org/
**YADAMP (7)**	Positive	https://webs.iiitd.edu.in/raghava/satpdb/catalogs/yadamp/
**DBAASP (8)**	Positive	https://dbaasp.org/home
**CAMP (9)**	Positive	http://www.camp3.bicnirrh.res.in/index.php
**AVPdb (10)**	Positive	http://crdd.osdd.net/servers/avpdb/
**Swiss-Prot (11)**	Negative	https://www.uniprot.org/

Supplementary Table3. 10-fold cross-validation results by AVP_training and AVP+GAN_training, respectively. Each fit is performed on a training set consisting of 90% of total training set at random, with the remaining 10% used as testing set for validation.

Training Set	Encoding method	Algorithm**	Accuracy	Precision	Sensitivity	Specificity	MCC
**AVP_training**	**PC6 encoding**	**CNN**	**0.886±0.012**	**0.876±0. 030**	**0.901±0.024**	**0.870±0. 042**	**0.773±0.022**
	PC6 encoding	RF	0.861±0.020	0.837±0.022	0.896±0.030	0.826±0.018	0.724±0.040
	PC6 encoding	SVM	0.842±0.018	0.811±0.035	0.889±0.014	0.795±0.027	0.686±0.034
	descriptor encoding*	CNN	0.861±0.018	0.857±0.021	0.866±0.024	0.856±0.021	0.722±0.036
**AVP+ GAN_training**	**PC6 encoding**	**CNN**	**0.939±0.005**	**0.935±0.014**	**0.944±0.011**	**0.934±0.015**	**0.878±0.009**
	PC6 encoding	RF	0.905±0.005	0.861±0.008	0.966±0.004	0.844±0.007	0.817±0.008
	PC6 encoding	SVM	0.934±0.004	0.919±0.007	0.952±0.005	0.916±0.007	0.868±0.008
	descriptor encoding*	CNN	0.875±0.007	0.869±0.008	0.883±0.007	0.866±0.011	0.750±0.013

Supplementary Table 4. The comparison of AVP predictors, trained with 2012_training, evaluated the performance using AVP_testing (293 positives, 293 negatives). There is no any overlap between AVP_testing and 2012_training.

Training dataset	Predictors	Accuracy	Precision	Sensitivity	Specificity	MCC
**2012_training***	AVPpred (1)	0.55	**0.61**	0.29	**0.82**	0.12
	AntiVPP1.0 (2)	**0.56**	0.58	0.44	0.68	0.13
	Meta-iAVP (3)	0.55	0.56	**0.49**	0.61	-0.04
	FIRM-AVP (4)	0.48	0.48	0.47	0.49	0.10
	AI4AVP ** (this study)	0.55	0.56	0.46	0.64	0.11

Remarks:

※ **AVP_testing**: 293 positives + 293 negatives, selected from a clean AVP collection in this study (AVP_fullset, 2,934 positives + 2,934 negatives).

*: 2012_training were collected by Thakur's work (1) for the AVPpred with 506 AVPs and 506 non-AVP.

**: we trained the same CNN model of AI4AVP with the 2012_training set.

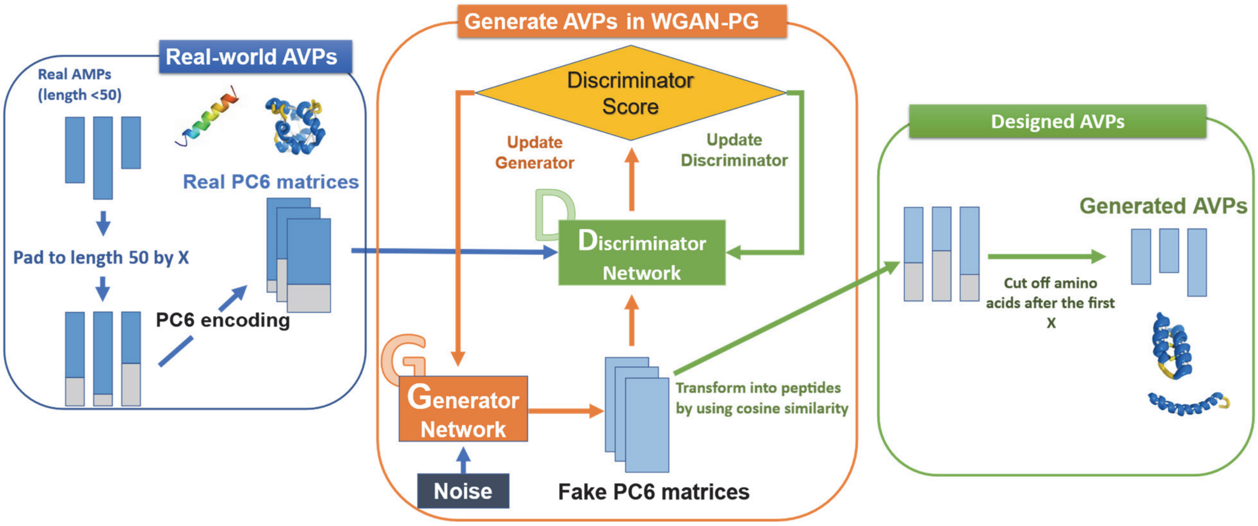

Supplementary Figure 1. The proposed GAN model for generating AVPs. Two neural networks, the *generator,* and the *discriminator,* are wrapped to form a backpropagation relationship. Briefly, the *generator* takes noise as its input to create AVP-like sequences (generated AVPs). The *discriminator* is trained to distinguish real AVPs from generated AVPs. The training aims to increase the generator's success rate by producing AVP-like peptides that fool the *discriminator*.

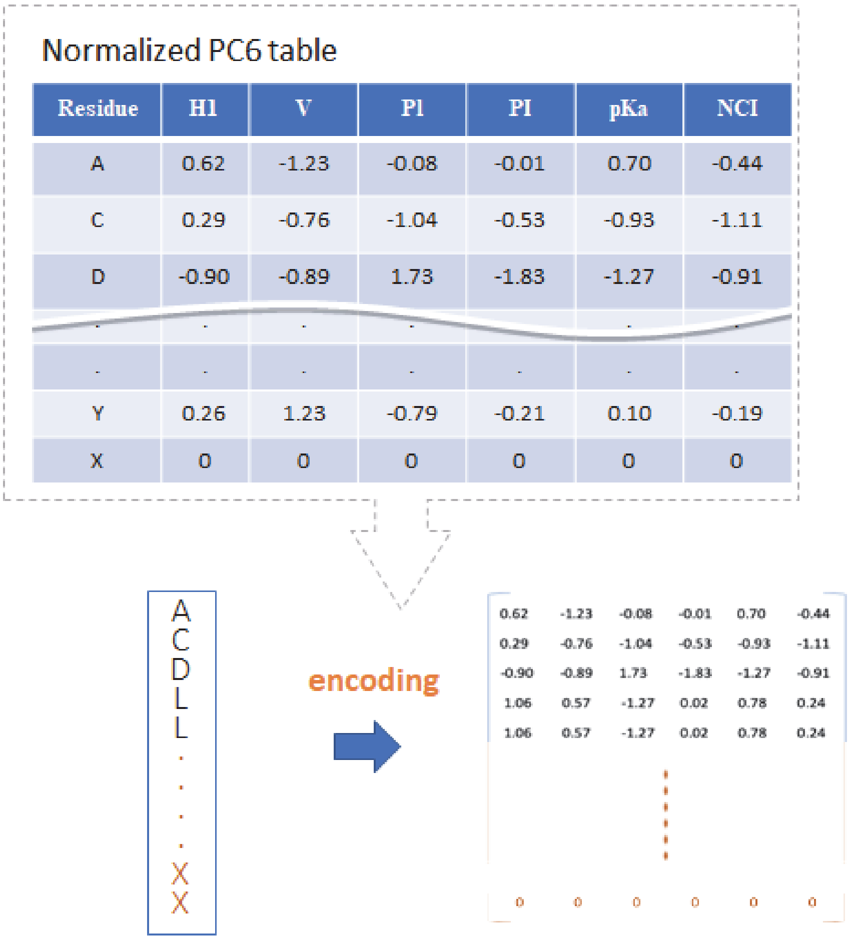

Supplementary Figure 2. The protein-encoding method PC6 is based on the physicochemical properties of each residue shown in the sequence. The normalized score of Hydrophobicity (H1), the volume of side chains (V), polarity (Pl), pH at the isoelectric point (pI), the dissociation constant for the -COOH group (pKa), and the net charge index of the side chain (NCI) are selected to present features of each residue component. The input peptide is padded with X if the length is shorter than 50 and is converted to a 50x6 matrix.

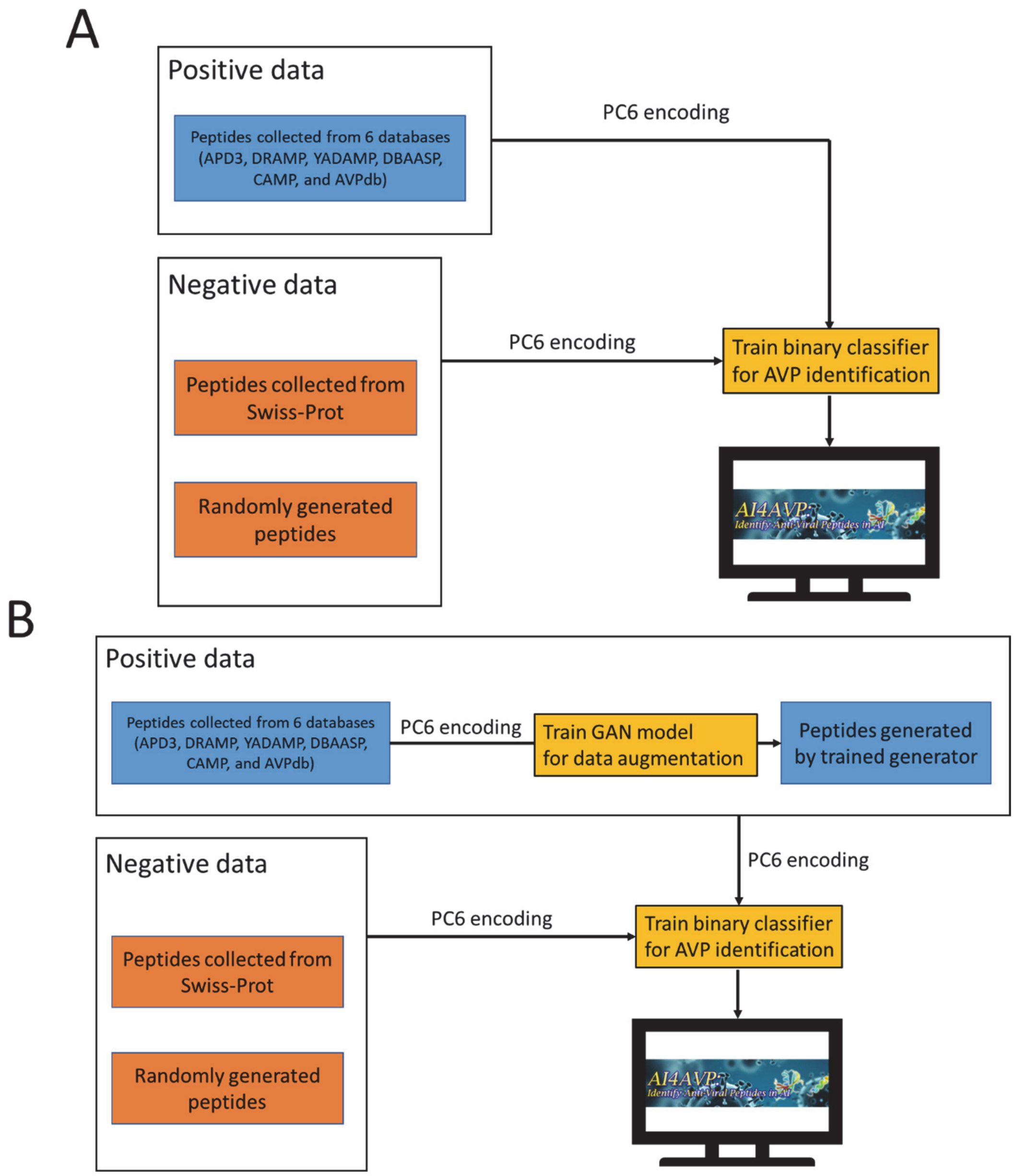

Supplementary Figure 3. The pipeline of AI4AVP development.
(A) The model trained on a positive dataset, AVP_training, consisting of real AVPs collected from AVP databases and an equal amount of non-AVP sequences. To make a balanced input in model training, the number of non-AVPs is limited. (B) The model was trained using AVP+GAN_training. We applied GAN for data augmentation; in this way, the positives consist of real AVPs plus GAN-generated AVPs, and almost all sequences in the negative data can be used for model training.

References

1. Thakur N, Qureshi A, Kumar M. 2012. AVPpred: collection and prediction of highly effective antiviral peptides. Nucleic acids research 40:W199-W204.

2. Lissabet JFB, Belén LH, Farias JG. 2019. AntiVPP 1.0: A portable tool for prediction of antiviral peptides. Computers in biology and medicine 107:127-130.

3. Schaduangrat N, Nantasenamat C, Prachayasittikul V, Shoombuatong W. 2019. Meta-iAVP: a sequence-based meta-predictor for improving the prediction of antiviral peptides using effective feature representation. International journal of molecular sciences 20:5743.

4. Chowdhury AS, Reehl SM, Kehn-Hall K, Bishop B, Webb-Robertson B-JM. 2020. Better understanding and prediction of antiviral peptides through primary and secondary structure feature importance. Scientific reports 10:1-8.

5. Li X, Wang Z, Wang G. 2015. APD3: the antimicrobial peptide database as a tool for research and education. Nucleic Acids Research 44:D1087-D1093.

6. Kang X, Dong F, Shi C, Liu S, Sun J, Chen J, Li H, Xu H, Lao X, Zheng H. 2019. DRAMP 2.0, an updated data repository of antimicrobial peptides. Scientific Data 6:148.

7. Piotto SP, Sessa L, Concilio S, Iannelli P. 2012. YADAMP: yet another database of antimicrobial peptides. International journal of antimicrobial agents 39:346-351.

8. Pirtskhalava M, Amstrong AA, Grigolava M, Chubinidze M, Alimbarashvili E, Vishnepolsky B, Gabrielian A, Rosenthal A, Hurt DE, Tartakovsky M. 2021. DBAASP v3: database of antimicrobial/cytotoxic activity and structure of peptides as a resource for development of new therapeutics. Nucleic Acids Research 49:D288-D297.

9. Waghu FH, Barai RS, Gurung P, Idicula-Thomas S. 2016. CAMPR3: a database on sequences, structures and signatures of antimicrobial peptides. Nucleic Acids Research 44:D1094-D1097.

10. Qureshi A, Thakur N, Tandon H, Kumar M. 2014. AVPdb: a database of experimentally validated antiviral peptides targeting medically important viruses. Nucleic acids research 42:D1147-D1153.

11. Boeckmann B, Bairoch A, Apweiler R, Blatter M-C, Estreicher A, Gasteiger E, Martin MJ, Michoud K, O'Donovan C, Phan I. 2003. The SWISS-PROT protein knowledgebase and its supplement TrEMBL in 2003. Nucleic acids research 31:365-370.
